# An *in-silico* comparison of proton beam and IMRT for postoperative radiotherapy in completely resected stage IIIA non-small cell lung cancer

**DOI:** 10.1186/1748-717X-8-144

**Published:** 2013-06-15

**Authors:** Abigail T Berman, Boon-Keng Kevin Teo, Derek Dolney, Samuel Swisher-McClure, Kambiz Shahnazi, Stefan Both, Ramesh Rengan

**Affiliations:** 1 Department of Radiation Oncology, University of Pennsylvania, Philadelphia, PA, USA; 2 Radiation Oncology, Seattle Cancer Care Alliance Proton Center, 1959 NE Pacific St, Box 356043, Seattle, WA, USA

**Keywords:** Proton, IMRT, Post-operative, NSCLC, Adjuvant, Dosimetry

## Abstract

**Introduction:**

Post-operative radiotherapy (PORT) for stage IIIA completely-resected non-small cell lung cancer (CR-NSCLC) has been shown to improve local control; however, it is unclear that this translates into a survival benefit. One explanation is that the detrimental effect of PORT on critical organs at risk (OARs) negates its benefit. This study reports an in-silico comparative analysis of passive scattering proton therapy (PSPT)- and intensity modulated proton therapy (IMPT) with intensity modulated photon beam radiotherapy (IMRT) PORT.

**Methods:**

The computed tomography treatment planning scans of ten patients with pathologic stage IIIA CR-NSCLC treated with IMRT were used. IMRT, PSPT, and IMPT plans were generated and analyzed for dosimetric endpoints. The proton plans were constructed with two or three beams. All plans were optimized to deliver 50.4 Gy(RBE) in 1.8 Gy(RBE) fractions to the target volume.

**Results:**

IMPT leads to statistically significant reductions in maximum spinal cord, mean lung dose, lung volumes treated to 5, 10, 20, and 30 Gy (V5, V10, V20, V30), mean heart dose, and heart volume treated to 40 Gy (V40), when compared with IMRT or PSPT. PSPT reduced lung V5 but increased lung V20, V30, and heart and esophagus V40.

**Conclusions:**

IMPT demonstrates a large decrease in dose to all OARs. PSPT, while reducing the low-dose lung bath, increases the volume of lung receiving high dose. Reductions are seen in dosimetric parameters predictive of radiation pneumonitis and cardiac morbidity and mortality. This reduction may correlate with a decrease in dose-limiting toxicity and improve the therapeutic ratio.

## Introduction

Outcomes following complete resection of stage IIIA non-small cell lung cancer (NSCLC) remain poor even with multimodality therapy. Post-operative radiotherapy (PORT) for stage IIIA completely-resected (CR) NSCLC has been previously shown to improve local control [1]; however, it is unclear that this improvement translates into a survival benefit. The PORT meta-analysis did not demonstrate an effect on survival in stage III patients [2]; a proposed explanation is that the detrimental effect is due to the use of large volumes, high doses, and older radiation techniques leading to injury to organs-at-risk (OARs), negating the clinical benefit [3]. Other studies have shown a survival benefit in CR-NSCLC patients with ipsilateral mediastinal and/or subcarinal nodal disease (defined as N2 or Stage IIIA disease) [4,5]. Standard indications for PORT in the modern era include multiple positive N2 nodes, high-risk pathologic features, or inadequate mediastinal surgical evaluation [6].

This study investigates the dosimetric benefit of proton therapy in decreasing the toxicity of PORT and therefore improving the therapeutic ratio. Intensity modulated radiotherapy (IMRT) has been shown to reduce dose to OARs and risk of pneumonitis [7]. Proton beam therapy (PBT), with its characteristic Bragg peak, holds the promise of further reducing toxicity. The radiation volume for PORT, the mediastinum, is an ideal target for PBT as it is located midline in the chest with many lymph node stations lying anteriorly; PBT has the potential to reduce radiation dose to the lungs, heart, esophagus, and spinal cord.

Several techniques exist for the administration of PBT, including passive scatter proton therapy (PSPT) and intensity modulated proton therapy (IMPT). While PSPT decreases dose distally, it is difficult to conform to a complex target such as the post-operative mediastinal volume, directly adjacent to critical structures such as esophagus, spinal cord, and heart. In contrast, IMPT uses scanning beam technology that modulates intensities of each pencil beam to take into account OAR constraints and target coverage [8].

This is the first report of the comparative dosimetric analysis of PSPT- and IMPT-PORT with IMRT- PORT for CR-NSCLC. Our objective was to assess whether these techniques reduce the dose to OARs when compared to IMRT, and to determine which treatment planning approach was superior.

## Materials and methods

### Study population

Ethical approval for this retrospective dosimetric study was obtained from the institutional review board at the University of Pennsylvania (number 808624) in compliance with the Helsinki Declaration. Ten patients with CR-NSCLC and treated with PORT at our institution from 2006 to 2008 were identified. Indications for PORT included positive margins and/or positive mediastinal and/or subcarinal lymph nodes. Five patients had right-sided tumors and five had left-sided. All patients were treated with 3D conformal radiotherapy (*n* = 5 patients) or IMRT (*n* = 5 patients).

### Definition of target volume

Patients underwent three-dimensional CT simulation in the supine position in a custom mold alpha cradle from the mandible to the iliac crest (3 mm slices). The clinical target volume (CTV) was defined as all mediastinal nodes, the ipsilateral hilum and the bronchial stump. Level 5 (aortopulmonary window) and level 6 (para-aortic) were only included for left sided tumors. Subcarinal (station 7) and paratracheal (station 4) lymph node stations were always included. Borders of lymph node stations were defined according to the CT-based guidelines published by Chapet *et al.*[9]. The planning target volume (PTV) was defined as the CTV plus a 5 mm margin. The target volumes were identical in proton and IMRT plans.

### Critical structure definition

The following critical normal structures were delineated on each planning CT scan: body, spinal cord (defined from 1 cm above the PTV to 1 cm below), heart (defined from apex to aortic valve), esophagus (defined from hypopharyngeal origin to gastroesophageal junction), contralateral lung, ipsilateral lung, and total lung minus the PTV.

### Dose prescription

IMRT and proton plans were optimized to deliver 50.4 Gy (RBE) in 1.8 Gy fractions (28 fractions total). For PBT, we utilized a generic relative biologic effectiveness (RBE) of 1.1 as the conversion factor to produce the effective dose for PBT from the physical dose in Gray, per the International Commission on Radiation Units (ICRU) Report 78. All doses are listed in Gy (RBE).

### Treatment planning

The IMRT, PSPT, and IMPT plans were generated using the Eclipse Treatment Planning System, Version 8.9 (Varian Medical Systems, Palo Alto, CA). All plans were designed by a single physicist experienced in lung and PBT to minimize variability. The IMRT plans used four (*n =* 1 patient) or five (*n =* 9 patients) co-planar 6-MV photon fields with a dynamic multileaf collimator (dMLC). The IMRT normal tissue constraints included the spinal cord and lung minus the PTV for each patient. Our planning goals were to provide adequate PTV coverage. The volume of the PTV receiving 95% of the dose was maintained to be at least 95%, while minimizing the dose delivered to the OARs. Plans were evaluated both by analyzing dose-volume histograms (DVHs) and qualitatively by visually inspecting dose distribution on axial CT.

For proton planning, in accordance with the report in ICRU 78, the PTV was defined relative to the CTV on the basis of lateral uncertainties alone in the range of 0.5 to 1.0 cm. Adjustments were made during the beam-design process to take into account differences, if any, between the margins needed to account for uncertainties along the beam direction (range uncertainties) and those included in the lateral uncertainty defined PTV [10]. The distal margins used in PSPT were calculated using 3.5% of the distal CTV depth plus 3 mm [10]. The margin was decreased in IMPT using 3.5% of the distal CTV depth plus 1 mm as there is no compensator.

For both PSPT and IMPT plans, either a two- or three-field proton plan (co-planar) was devised in the following combinations to treat the PTV: left anterior oblique (LAO)/right anterior oblique (RAO), LAO/anterior posterior (AP), RAO/AP, or RAO/LAO/AP.

For PSPT plans, MLCs were designed to shape each field laterally, and a compensator was used to shape the distal portion of the beam to the PTV. To account for uncertainties in patient positioning, internal motion, and to ensure sufficient target coverage and proton scattering, the compensator smearing with a margin of approximately 1 cm was applied in accordance with the method described by Moyers *et al*. A 1 cm border-smoothing margin was applied to the compensator in order avoid protons traversing along the compensator wall outside the CTV which is not shielded by the aperture (MLC in our case) [10].

For IMPT plans, two to three fields were utilized to achieve optimum dose coverage to the PTV and dose sparing to the OARs. Optimization was performed by the planner adjusting the dose, volume, and penalty of each objective including targets and OARs. The range of energies needed for each field was calculated using the proximal and distal margins of the target structure. All available preconfigured energy layers within this range were used in the optimization.

The pencil beam data used in the optimization were not measured beam data (unavailable at time of study), but instead calculated data using Geant4-based Monte Carlo simulation that was customized to match vendor (IBA Particle Therapy, Belgium) specifications. Monte Carlo was utilized as it provides a more realistic beam data specific to our proton system compared to a generic beam dataset derived from other facilities.

The spot positions within each energy layer are fixed and the spot spacing is two-thirds of the full-width-at-half-maximum for the beam in air at isocenter. The number of monitor units (MU) to deliver for each spot was then determined using the simultaneous spot optimization algorithm [11]. The raw spot weights were then post-processed to adapt to the treatment machine limitations, especially those relating to the minimum number of MUs deliverable per spot, and the resolution of the monitor chambers responsible for measuring the MU per spot. During post-processing, the cut-and-append repainting strategy was utilized utilized whereby the dose per painting per energy layer was limited to 0.1 Gy. Repainting is recommended for scanned pencil beam delivery to mitigate any interplay effects between the motion of the beam and the motion of the target [12]. The final dose distribution and DVHs were calculated using the post-processed spot weights.

### DVH and statistical analysis

DVHs were analyzed for all of the OARs identified within the radiation fields. PSPT and IMPT plans were each compared to the IMRT plan and PSPT was compared directly to IMPT. The paired t-test was used for all statistical comparisons, with *p* values less than 0.05 considered significant. *P* values less than 0.001 were truncated and noted as *p* < 0.001.

### IMPT plan robustness

Assessment of the effect of setup errors on one IMPT plan was performed by recalculating the dose distributions with simulated ±3 mm shifts in the three orthogonal directions for this patient group. The nominal DVH statistics from the IMPT plan were then compared with the DVH band resulting from the simulated setup errors.

## Results

### OAR comparison of PSPT vs IMRT for PORT

**Figure 1:**
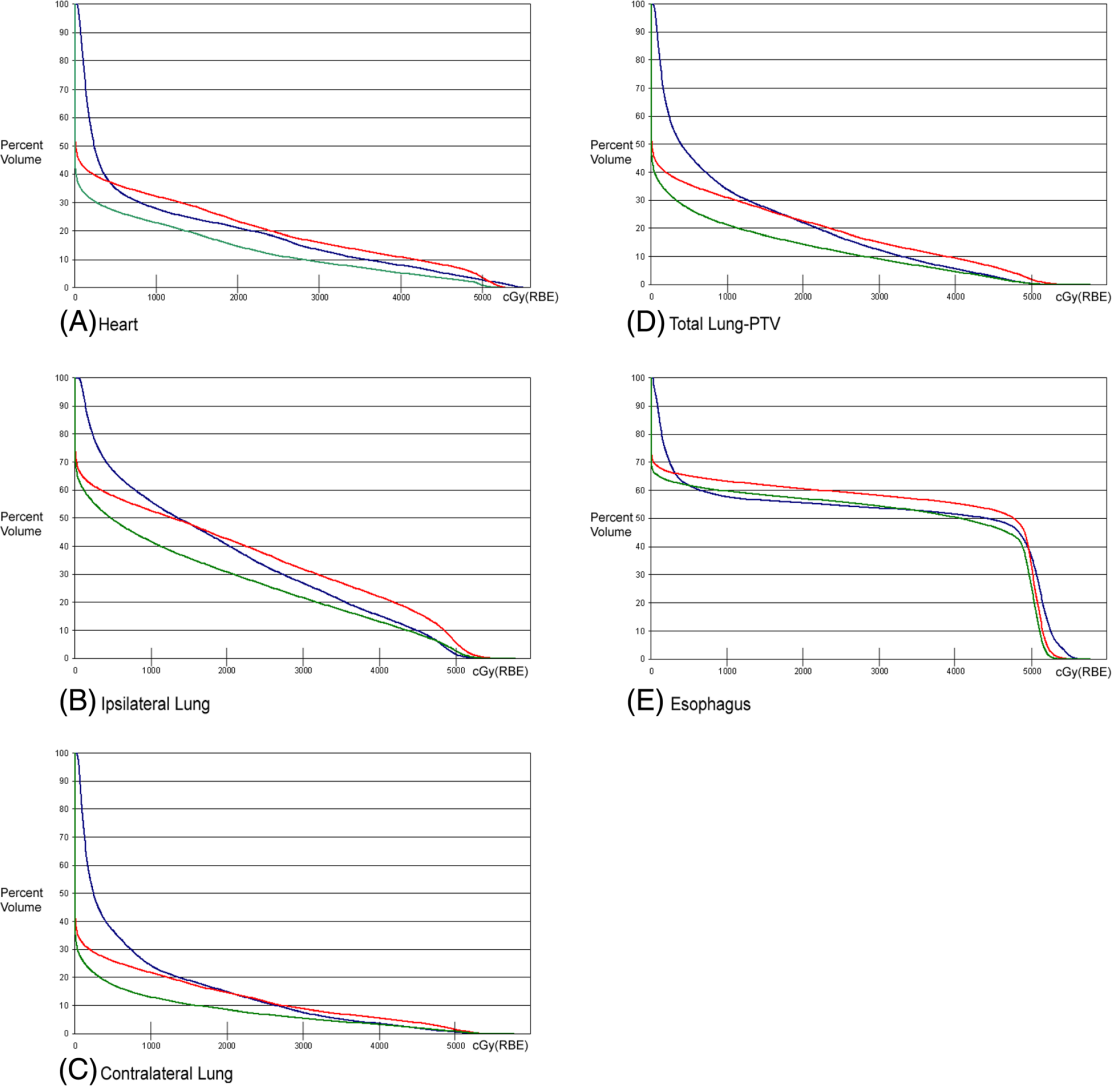
Average DVHs of 10 patients comparing IMRT (blue), PSPT (red), and IMPT(green), (A) heart, (B) ipsilateral lung, (C) contralateral lung, (D) total lung-PTV, (E) esophagus.

Compared with IMRT plans, PSPT plans spared the lung dose, however with a concomitant increase in the esophageal and heart doses (Table 1). The mean esophageal dose increased by 4.6% and the volume received by 40 Gy (V40) increased by 7.3% with PSPT versus IMRT. There was no significance difference in the mean heart dose however the heart V40 was significantly higher with PSPT versus IMRT (36%, *p* = 0.03). Figure 1 shows the average DVHs for all 10 patients for each organ at risk. There was no significant difference between PSPT and IMRT in the maximum external dose received (*p =* 0.7). PSPT produced a 14% reduction in the maximum cord dose compared with IMRT.

**Table 1:** Dosimetric endpoints for IMRT, PSPT, and IMPT plans including average values, standard deviation, and range

	***Parameter (%)***	**IMRT**				**PSPT**				**IMPT**			
		***Average***	***SD***	***Minimum***	***Maximum***	***Average***	***SD***	***Minimum***	***Maximum***	***Average***	***SD***	***Minimum***	***Maximum***
Total lung	V5	46.2	6.9	37.4	58.8	35.6	11.5	16.7	57.2	26.9	6.6	17.4	36.6
	V10	33.8	7.2	21.2	43.5	30.9	10.2	13.8	49.0	21.2	5.1	12.9	28.9
	V20	22.0	5.9	9.1	29.4	22.6	7.6	10.1	36.2	14.4	3.7	7.5	19.8
	V30	12.3	3.9	3.9	16.1	15.0	4.4	6.7	21.4	9.1	2.3	3.9	12.3
	Mean (cGy)	1083.0	212.8	686.0	1370.5	1003.6	306.8	475.1	1484.1	667.5	160.5	372.2	907.6
Contralateral lung	V5	36.7	11.8	24.8	65.8	25.9	14.0	7.3	52.6	17.3	11.1	4.0	42.5
	V10	24.2	13.3	9.7	56.9	21.7	13.0	5.0	47.4	13.0	9.6	2.5	36.2
	V20	14.8	10.8	1.6	41.1	14.6	10.9	2.4	37.0	8.5	7.9	1.1	28.6
	V30	7.6	8.5	0.3	30.4	8.8	7.7	1.0	28.0	5.5	6.1	0.3	21.7
	Mean (cGy)	813.1	434.1	404.8	1935.7	673.8	459.3	140.5	1707.0	428.2	351.8	72.7	1313.0
Ipsilateral lung	V5	67.0	13.5	45.0	89.1	57.9	19.3	18.1	84.9	49.5	15.1	11.6	64.5
	V10	55.9	13.0	33.0	76.4	52.7	17.9	15.5	76.2	41.6	13.6	8.7	55.3
	V20	40.5	13.9	14.5	58.5	42.5	14.7	10.4	56.6	30.8	11.2	5.2	41.5
	V30	26.8	11.8	3.7	44.5	31.8	11.6	6.7	43.6	21.6	8.8	3.2	32.9
	Mean (cGy)	1827.5	481.3	902.1	2482.6	1869.9	631.4	485.8	2504.0	1390.3	471.6	270.0	1869.9
Spinal cord	Dmax (cGy)	4018.9	482.5	2777.0	4480.5	3435.9	1096.5	1057.6	4699.6	1354.9	721.9	374.6	2530.6
	D1 (cGy)	3281.8	1348.7	80.1	4155.0	2413.4	1182.0	257.0	4364.0	817.6	163.0	515.4	1686.0
Heart	V40	7.9	7.0	0.1	23.4	10.8	6.0	1.2	19.0	5.2	4.6	0.2	14.5
	Mean (cGy)	1019.8	477.4	264.7	1954.0	1063.0	399.2	368.1	1462.0	688.9	316.7	76.3	1101.5
Esophagus	V40	51.5	11.6	30.0	73.7	55.3	10.4	38.6	74.5	50.3	12.4	32.0	74.4
	Mean (cGy)	2854.3	616.6	1878.2	4184.2	2985.8	575.9	2074.7	4056.0	2764.3	598.6	1888.3	3997.0
Body	Dmax (cGy)	5662.3	82.0	5495.7	5753.2	5688.2	171.7	5237.3	5878.6	5635.2	139.1	5410.9	5805.7

Abbreviations: *Dmax* maximal dose received, *D1* dose received by 1% (of the spinal cord).

**Figure 2:**
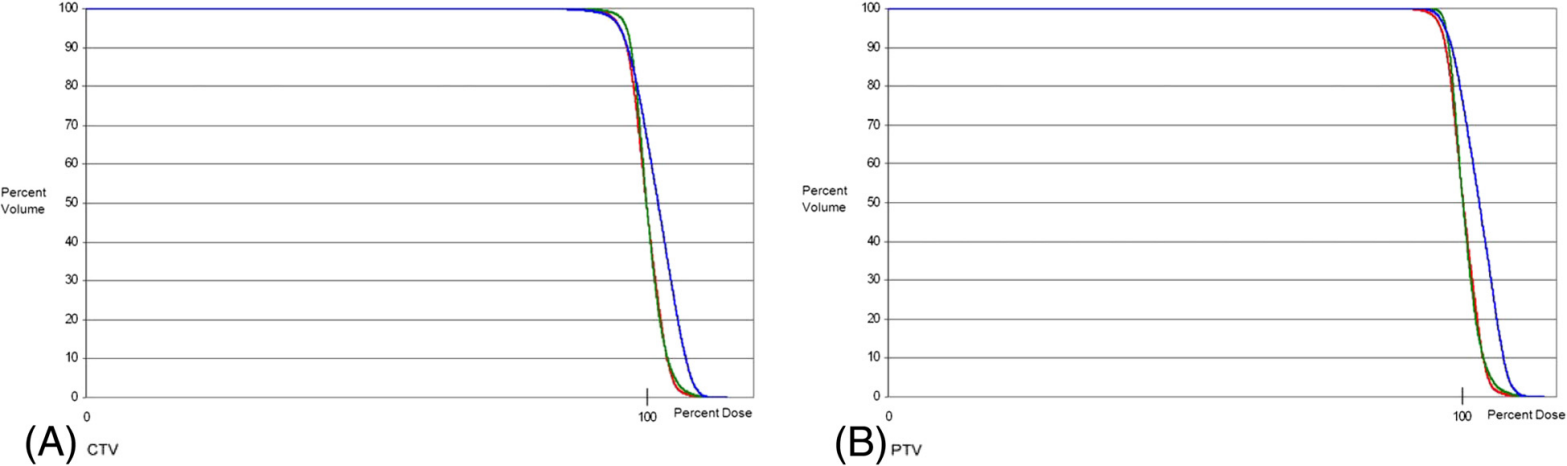
Average DVHs of 10 patients comparing IMRT (blue), PSPT (red), and IMPT (green) for the target structures: (A) PTV and (B) CTV.

There was an overall reduction in the lung volume that received low dose, however an increase in the lung volume received by high dose with PSPT (Table 2). Figures 2B-D show PSPT (red) yielding a higher volume irradiated at the high-dose regions compared with IMRT or IMPT.

**Table 2:** Comparison of PSPT vs. IMRT, IMPT vs. IMRT, and IMPT vs. IMRT for three lung volumes: total lung (minus PTV), contralateral lung, and ipsilateral lung

		**PSPT vs. IMRT**		**IMPT vs. IMRT**		**IMPT vs. PSPT**	
		***∆ (%)***	***p value***	***∆ (%)***	***p value***	***∆ (%)***	***p value***
Total lung	V5	−22.8	0.002	−41.7	<0.001	−24.5	0.001
	V10	−8.6	0.2	−37.3	<0.001	−31.4	<0.001
	V20	2.9	0.7	−34.5	<0.001	−36.4	<0.001
	V30	22.1	0.005	−26.2	0.001	−39.6	<0.001
	Mean (cGy)	−7.3	0.2	−38.4	<0.001	−33.5	<0.001
Contralateral lung	V5	−29.4	<0.001	−52.8	<0.001	−33.1	<0.001
	V10	22.1	0.006	−26.2	0.001	−39.6	<0.001
	V20	−1.7	0.8	−42.5	<0.001	−41.5	0.002
	V30	15.7	0.009	−27.5	0.03	−37.3	<0.001
	Mean (cGy)	−17.1	0.006	−47.3	<0.001	−36.4	<0.001
Ipsilateral lung	V5	−13.5	0.02	−26.1	<0.001	−14.6	0.007
	V10	−5.8	0.3	−25.6	<0.001	−21.0	<0.001
	V20	5.1	0.4	−23.9	<0.001	−27.6	<0.001
	V30	18.8	0.007	−19.4	0.009	−32.1	<0.001
	Mean (cGy)	2.3	0.6	−23.9	<0.001	−25.6	<0.001

Delta represents change of first technique listed versus baseline comparison technique (second listed).

### OAR comparison of IMPT vs IMRT for PORT

IMPT plans reduced the mean esophageal dose by 3.2% and there was no significant change in V40. IMPT significantly reduced the mean heart dose by 30.7% over IMRT and there was a trend towards a lower V40 with IMPT (Table 1).

All lung doses were consistently reduced with IMPT over IMRT (Table 2).

### OAR comparison of IMPT vs PSPT for PORT

IMPT plans reduced the mean esophageal dose by 7.4% and the V40 by 9% versus PSPT. The mean heart dose and V40 were significantly reduced by 35.1% (*p <* 0.001) and 51.7% (*p <* 0.001), respectively. There was no significant difference between IMPT and PSPT in the maximum external dose received (*p =* 0.3). In addition, IMPT reduced the maximum cord dose by 60% over PSPT (*p* < 0.001*).* There was also a significant reduction in the dose received by 1% of the spinal cord volume, including a 66% reduction with IMPT over PSPT (Table 1).

IMPT yielded large and statistically significant reductions in all lung doses over PSPT (Table 2). The magnitude of benefit of IMPT over PSPT was larger in the volume receiving higher doses.

### CTV coverage comparison with IMPT, PSPT, and IMRT

All plans maintained adequate coverage of the PTV with V95 at least 95%. The average V95 for IMRT, PSPT, and IMPT were 96.0%, 96.2%, and 98.4%, respectively. CTV and PTV coverage were similar among all three groups, as shown in Figure 2.

**Figure 3:**
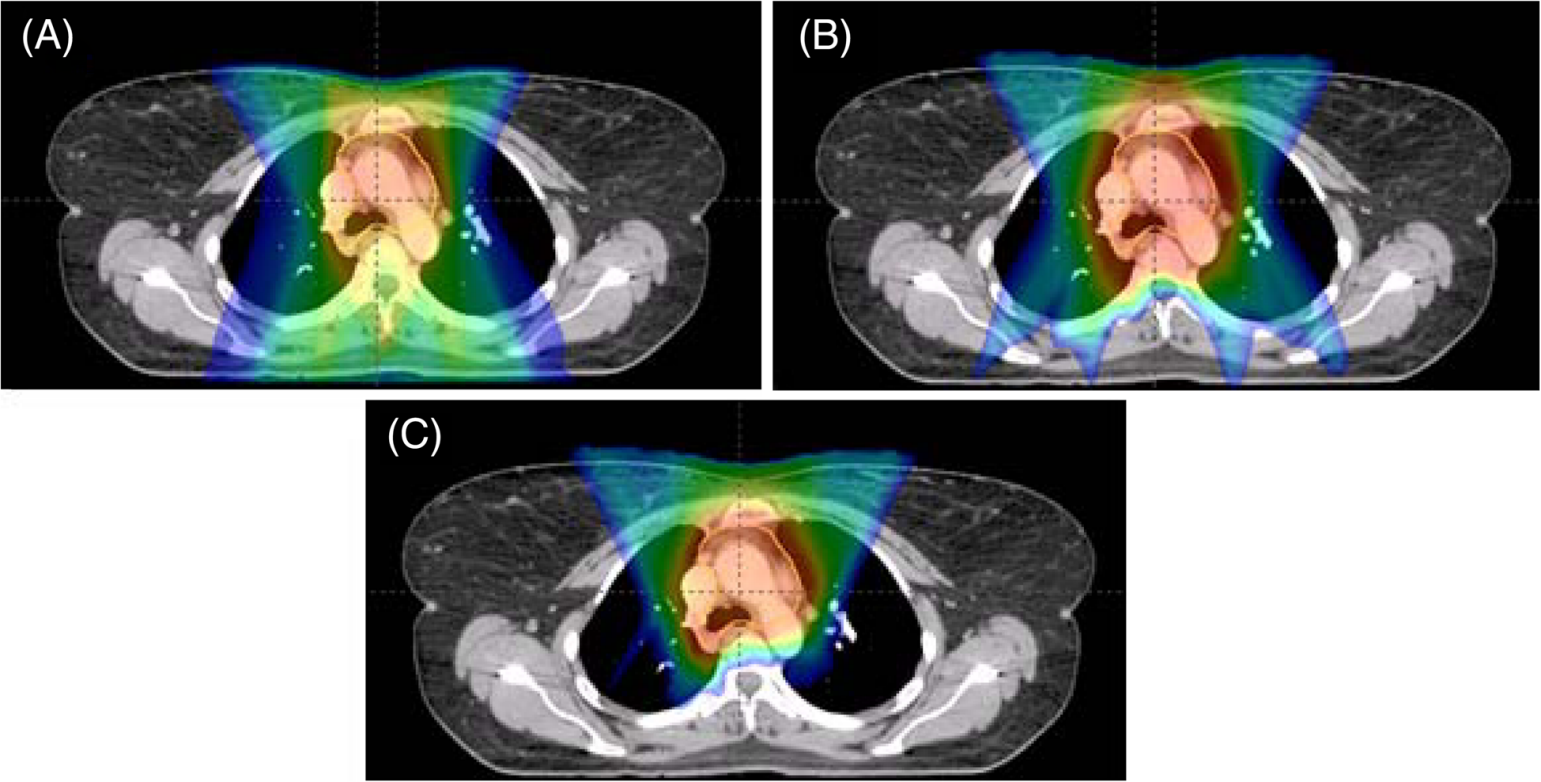
**Patient with stage IIIA NSCLC status post right upper lobe lobectomy with positive lymph nodes in station 7.** Representative isodose distributions for (**A**) IMRT (**B**) PSPT (**C**) IMPT plans in the axial view. Key demonstrates corresponding dose to colorwash.

**Figure 4:**
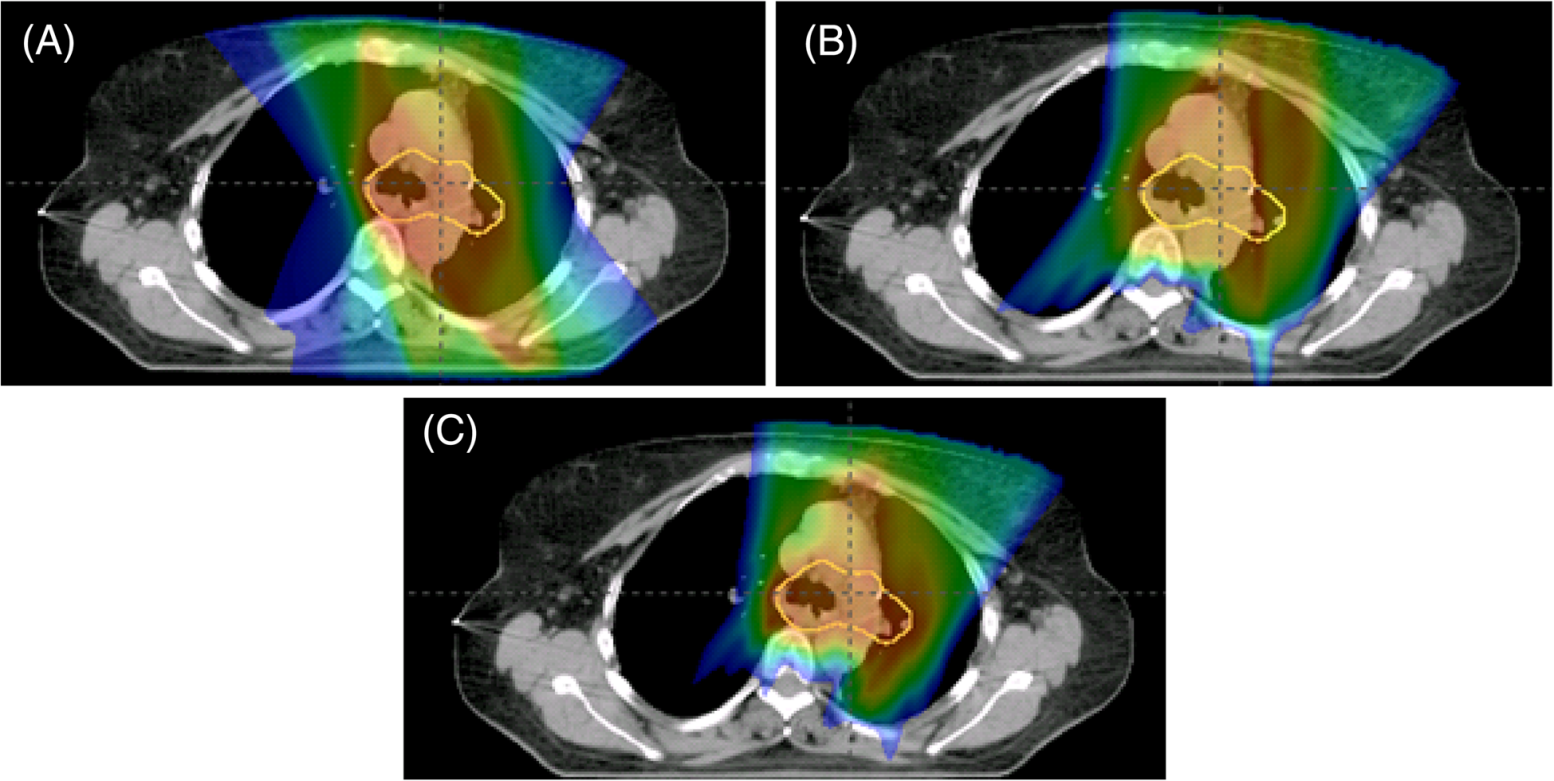
**Patient with stage IIIA NSCLC status post left lower lobe lobectomy with positive lymph nodes in stations 5 and 12.** Representative isodose distributions for (**A**) IMRT (**B**) PSPT (**C**) IMPT plans in the axial view. Key demonstrates corresponding dose to colorwash.

Figures 3 and 4 show patients with stage IIIA NSCLC status post right upper lobe lobectomy with positive lymph nodes in station 7 and status post left lower lobe lobectomy with positive lymph nodes in stations 5 and 12, respectively. In both cases, the dosimetric benefit of IMPT can be qualitatively appreciated. It is noted that the 4500 cGy isodose line approaches the anterior thoracic skin in the PSPT plan but generally spared to lower doses in the IMRT or IMPT plans.

### Uncertainty in IMPT plan delivery

**Figure 5:**
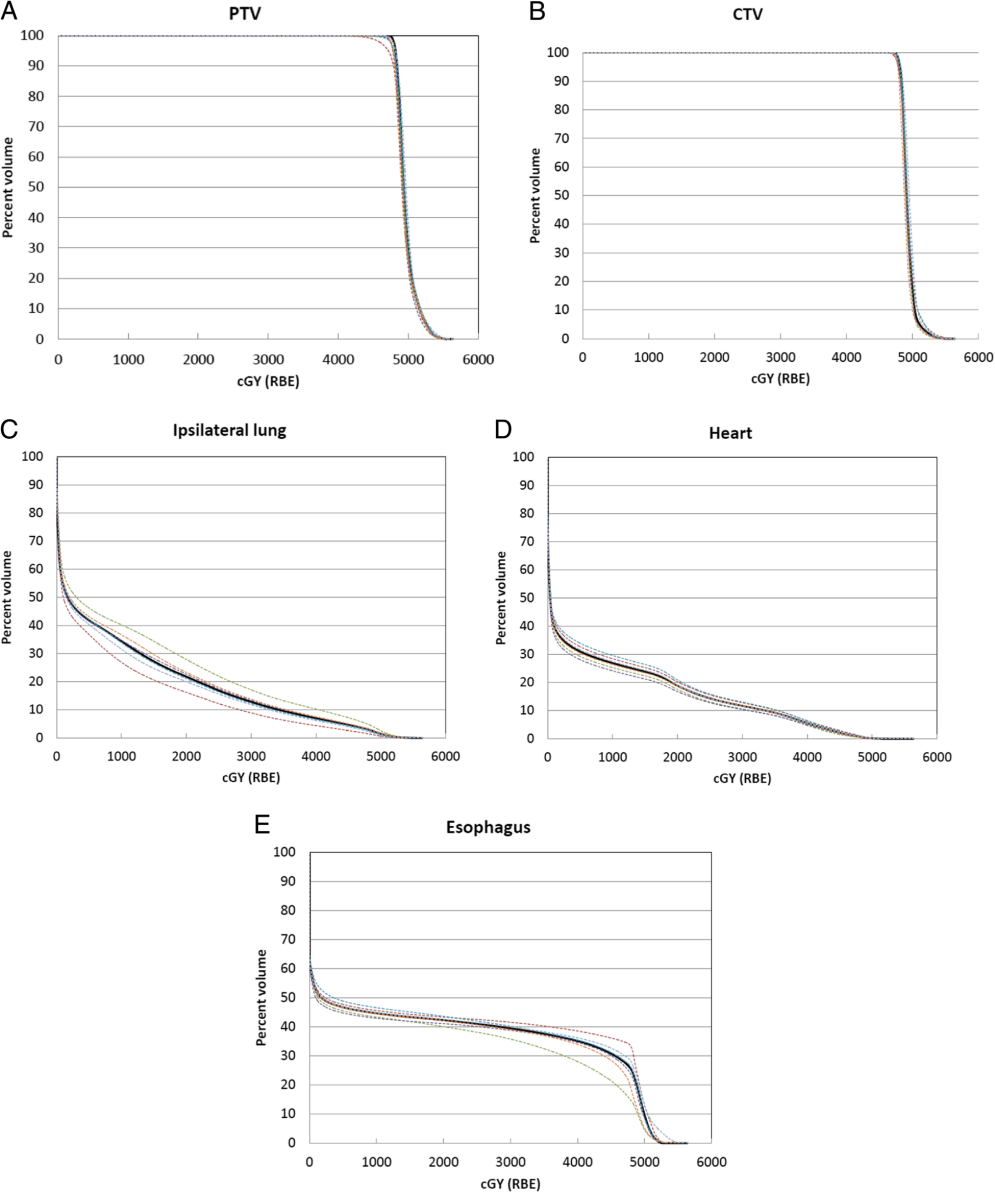
**DVH bands of the targets (A), (B) and organ at risks (C)-(E).** The nominal DVH is shown as the black line while dotted lines are results from lateral, vertical and longitudinal setup errors of 3mm. The worst case DVH results from lateral setup errors.

Since the treatment fields were mostly anterior, lateral setup errors resulted in the largest deviation in dose to the target from the nominal values. For the illustrative case, PTV V95% decreased from 98.1% to 90.2% (Figure 5A) while CTV V95% decreased from 97.5% to 94.5% (Figure 5B). For the ipsilateral lung (Figure 5C), V20 increased from a nominal value of 21.7% to a maximum of 27.9% as a consequence of beam overshoot in the lungs due to setup errors. For the heart (Figure 5D), V40 increased from a nominal value of 5.5% to a maximum of 6.3%.

## Discussion

We demonstrated that IMPT decreases the dose to all OARs versus both IMRT and PSPT. This decrease in dose to OARs theoretically corresponds to a decrease in the potentia radiation morbidity. Based on this dosimetric study, proton beam therapy has the potential to provide adjuvant radiotherapy that is both safe and efficacious to patients with CR-NSCLC.

It is hypothesized that the lack of survival benefit seen in some PORT studies is due to the adverse effects of older radiation techniques. In 1998, the PORT meta-analysis showed a survival decrement for all patients, but not for patients with N2 disease [2]. A retrospective subset analysis of the Adjuvant Navelbine International Trialist Association (ANITA) randomized study of adjuvant chemotherapy found that for patients with pN2 disease, PORT improved median survival in patients who received chemotherapy (23.8 to 47.4 months) and in those who did not receive chemotherapy (12.7 to 22. 7 months) [5]. A SEER analysis of PORT found a survival benefit (hazard ratio of 0.855) for N2 patients on subset analysis. The radiotherapy in these studies included Cobalt 60 and two-dimensional treatment planning. This type of radiotherapy led to excess heart and lung dose corresponding to both short- and long-term morbidity and mortality. However, using modern radiotherapy techniques, the risk of death from intercurrent disease (DID) is not excessively increased, as shown by Machtay *et. al.* (13.5% with PORT versus 10%) [13].

PBT has the potential to decrease dose to OARs above standard modern radiotherapy and, therefore, improve the therapeutic index [14]. Other groups have shown superiority of PSPT over 3D-CRT [15] as well as IMPT over PSPT in the definitive treatment of NSCLC [16]. There are two unique aspects of post-operative radiotherapy that make proton beam therapy a particularly well-suited treatment. First, unlike in the definitive setting, the target structure is midline and anterior without extension into the lung parenchyma. This is easily approached with two-or-three field beam configurations. Alternatively, an incline gantry arrangement may allow for a non-coplanar beam arrangement which could decrease the dose to the heart even further. Second, in definitive treatment, the therapeutic index is inherently wider since radiotherapy is the only method of local control whereas, in PORT, surgery is the primary treatment and radiotherapy is given adjuvantly. Therefore, the risk of adverse effects with PORT must be smaller than with definitive RT and PBT could help minimize these risks.

Radiation-induced lung injury (RILI), including pneumonitis and pulmonary fibrosis, is the most significant contributor to the morbidity and mortality of PORT. We demonstrate IMPT decreases lung doses of up to 41% versus PSPT and up to 53% versus IMRT. While PSPT reduced the low-dose lung bath versus IMRT, there was an increase in the high-dose volume irradiated.

The clinical implications of these findings are that IMPT may decrease the incidence of RILI. Dose-volume relationships including the MLD, V5, V15 and V20 have been correlated with risk of pneumonitis [17-19]. The development of RILI also depends on field size [3], use of chemotherapy, baseline pulmonary function, and genetic polymorphisms in the ATM-P53 and base excision repair (XRCC1,APEX1) pathways [20]. IMPT, shown to reduce all examined lung parameters, should reduce RILI; it is unclear if PSPT would also lead to a clinically significant decreased incidence of RILI.

PORT has been shown to increase heart disease mortality, although not when examining patients treated with modern radiotherapy techniques [21]. We show a large reduction in mean heart dose and V40 with IMPT versus IMRT and PSPT but an increase with PSPT over IMRT, likely from an improved ability of IMPT to conform to a complicated CTV. Given the concern for heart morbidity, we recommend that caution be taken when treating the mediastinum with PSPT. Although IMPT treatment planning shows promising results in terms of RT-induced cardiac toxicity, motion management and repainting delivery techniques should be employed in order to ensure accurate delivery of the treatment plan.

Proton planning has inherent uncertainties, including inhomogeneity. In PSPT, as the beam passes through a low density structure (e.g. lung), its distal edge is degraded. With IMPT, this degradation is limited and the distal margin can be truncated accordingly as the distal target pencil beam energies and intensities are optimized to cover the distal target without degradation. In PSPT, the compensator is smeared to account for setup uncertainties and motion to ensure target coverage. This can have dramatic effect in the distal dose distribution for lung treatment due to the presence of lung tissue in the beam path. In IMPT, setup uncertainties and motion can also have a dramatic effect on dose homogeneity. The magnitude of these errors depends on the internal dose gradients as well as the pencil beam widths [22]. Active motion management, with 4D CT implemented as either prospective gated imaging or retrospective correlative imaging may be a way to reduce some of these uncertainties and further clinical investigations are necessary to confirm this.

Robustness of IMPT plans against setup and range uncertainties is a topic of active research [23]. In particular, setup errors lateral to the beam direction may result in large overshoot into the surrounding lung tissue, negating any potential lung benefit that IMPT may offer over other modalities. In PSPT, this effect is taken into account in the planning process through compensator smearing. For IMPT, Pflugfelder [24] and Liu [25] have proposed incorporating geometrical and range uncertainties within the robust optimization framework by taking into account the worst case dose distribution in the objective function, thereby ensuring that the target dose is more homogeneous and OAR doses are less sensitive to these uncertainties. In this work, we presented one illustrative case of the uncertainties in the IMPT dose distribution arising from setup errors. The results indicated that PTV coverage may drop by 8% and the CTV coverage by 3%. OAR doses are also sensitive to setup uncertainties but the nominal DVHs tends to lie in the middle of the DVH bands.

Another uncertainty with PBT is inter- and intra-fraction organ and target motion. While motion of the mediastinum is less than a distal lung tumor, it is nonetheless present. At our institution, we account for this with 4D simulation for all patients who undergo thoracic radiotherapy (PSPT), as studies have shown that by accounting for motion at the time of simulation, CTV coverage is adequate throughout radiotherapy [26]. Issues of dynamic delivery and tumor motion are more significant with IMPT than PSPT; dose repainting with fractionated IMPT and active motion management even for small amplitude motion may be necessary. Further studies need to be performed to examine this interplay effect which has been shown to cause a possible difference in dose of up to 10% [27] but can be mitigated by beam rescanning.Patients have been treated at our institution with PSPT to the mediastinum, and we have found the same dosimetric benefit as is demonstrated in this study when motion was taken into consideration. As technology with IMPT further advances, we anticipate a similar dosimetric benefit with motion consideration as seen in this study.

Other notable findings include a decrease in esophageal and cord doses by IMPT over PSPT. The changes in magnitude are smaller than seen in the heart and lung doses, largely due to the esophagus abutting or even being within the PTV. Nonetheless, this reduction in dose may correspond to a decrease in treatment-related esophagitis, a dose-limiting acute toxicity. There were significant reductions in the cord dose. Although clinically-significant cord toxicity is rare in NSCLC, this large reduction could permit dose-escalation in cases of positive margins and gross residual disease. It is notable that the skin dose is higher with proton therapy than IMRT, which could result in greater acute skin toxicity and erythema. Overall, the long-term potential benefits of IMPT on the lung and heart outweigh its possible increased skin toxicity which resolves shortly after radiotherapy.

We utilized DVH constraints identical to those used at our institution for proton and IMRT plans. All contouring was performed by a single physician (ABM) and a single physicist (KT) performed all ten treatment plans to minimize designer bias.

Secondary particles including neutrons may theoretically deliver dose outside of the target volume (in PSPT, from the treatment nozzle; in IMPT, from interactions with nuclei of tissue). There is no published evidence of an increase in secondary malignancies from neutron dose from PBT; however, this remains an area of uncertainty in our calculations and long-term effects.

In summary, compared with IMRT and PSPT, IMPT had a statistically significant and large decrease in dose to all OARs, most notably the lung and heart, all while maintaining excellent target coverage. PSPT, while reducing the volume of lung receiving a higher dose, increases the low-dose bath and increases certain heart dosimetric parameters. The clinical implications are that IMPT will hopefully reduce the adverse effects of PORT and improve the therapeutic index when employed in conjunction with active motion management and repainting techniques.

At the University of Pennsylvania, there are ongoing phase II trials utilizing proton beam therapy in the definitive setting for NSCLC. At MD Anderson Cancer Center and Massachusetts General Hospital, there is a randomized trial of proton versus photon therapy for locally advanced lung cancer. In the post-operative setting, phase I/II trials are necessary to correlate the clinical significance of the finding of this study and clarify the role of IMPT in the post-operative treatment of NSCLC. Subsequently, a randomized phase III trial could be designed comparing proton and photon therapy for PORT of resected NSCLC with a primary endpoint of reducing cardiopulmonary toxicity.

## Consent

An ethical waiver was obtained for publication of this report and any accompanying images.

## Competing interests

We do not have any conflicts or potential conflicts of interest to disclose at this time. We do not use any copyrighted information or patient photos. The data presented in this manuscript was acquired in accordance with the policies of the Institutional Review Board at the University of Pennsylvania. One of the authors (DD) was supported by the US Army Medical Research and Materiel Command under Contract Agreement No. DAMD17-W81XWH-04-2-0022. Opinions, interpretations, conclusions and recommendations are those of the authors and are not necessarily endorsed by the US Army.

## Authors' contributions

ATB participated in the design of the study, analysis of data, and preparation of manuscript. BKT participated in the design of the study, physics planning, and preparation of manuscript. DD participated in the physics planning and preparation of manuscript. SSM participated in the preparation of manuscript. KS participated in the design of the study and physics planning. SB participated in the design of the study and physics planning. RR participated in the design of the study, analysis of data, and preparation of manuscript. All authors read and approved the final manuscript.
